# Association of explanatory histological findings and urinary protein and serum creatinine levels at renal biopsy in lupus nephritis: a cross-sectional study

**DOI:** 10.1186/s12882-020-01868-9

**Published:** 2020-06-01

**Authors:** Eri Katsuyama, Yoshia Miyawaki, Ken-ei Sada, Yosuke Asano, Keigo Hayashi, Yuriko Yamamura, Sumie Hiramatsu-Asano, Michiko Morishita, Keiji Ohashi, Haruki Watanabe, Takayuki Katsuyama, Mariko Narazaki, Yoshinori Matsumoto, Jun Wada

**Affiliations:** 1 Department of Nephrology, Rheumatology, Endocrinology and Metabolism, Okayama University Graduate School of Medicine, Dentistry and Pharmaceutical Sciences, 2-5-1 Shikata-cho, Kitaku, Okayama, 700-8558 Japan; 2 Department of Healthcare Epidemiology, School of Public Health in the Graduate School of Medicine, Kyoto University, Kyoto, Japan

**Keywords:** Lupus nephritis, Active lesions, Chronic lesions, Urinary protein, Serum creatinine

## Abstract

**Background:**

The aim of the present study was to evaluate the association between the histology of active and chronic lesions and urinary protein and serum creatinine (SCr) levels, as common clinical endpoints in clinical trials for lupus nephritis (LN).

**Methods:**

In total, 119 patients diagnosed with LN class III, IV, and V, as defined by the International Society of Nephrology/Renal Pathology Society, between 1990 and 2015, were enrolled in the present study. Multiple regression analysis was performed to explore semi-quantitative histological variables associated with urinary protein and SCr levels.

**Results:**

The mean age of the enrolled patients was 45 years, and 79% were female. The mean SCr and mean urinary protein levels at the time of renal biopsy were 0.87 mg/dl and 3.00 g/gCr, respectively. Class IV (71%) was the most common type of LN followed by class III (17%), and class V (13%). Multicollinearity was confirmed between monocellular infiltration (variance inflation factor [VIF] = 10.22) and interstitial fibrosis (VIF = 10.29), and between karyorrhexis (VIF = 4.14) and fibrinoid necrosis (VIF = 4.29). Fibrinoid necrosis and monocellular infiltration were subsequently excluded, and multiple regression analysis revealed that only the urinary protein level was correlated with wire loop lesions (β-coefficient [β]: 1.09 and confidence interval [CI]: 0.35 to 1.83), and that the SCr level was correlated with glomerular sclerosis (β: 1.08 and CI: 0.43 to 1.74).

**Conclusion:**

As urinary protein and SCr levels were not quantitatively associated with active lesions, they may not accurately reflect the response to remission induction therapy in patients with LN.

## Background

While clinical trials for promising therapeutic agents for lupus nephritis (LN), such as B cell targeted therapy, cytokine-targeted therapy (IL-6 and IFN-a), and cytotoxic T lymphocyte-associated antigen 4, have been conducted, none have shown improved outcomes compared with controls [1]. These failures may be attributed to inadequate inclusion criteria, study populations, sample sizes, and study duration [1, 2]. Furthermore, one study focused on the definition of outcome measurements [3, 4].

Proteinuria and serum creatinine (SCr) have been considered as promising predictors for renal prognosis in patients with LN [5–8]. Consequently, the primary outcome of the majority of clinical studies for LN is defined by urinary protein and SCr levels [9, 10]. Previous studies have revealed that measures of chronic lesions in LN, such as the chronic index, glomerular sclerosis, and interstitial fibrosis, are related to a poor renal outcome [11, 12]. The estimated glomerular filtration rate (eGFR) is calculated using the SCr level, and eGFR and proteinuria are biomarkers for the classification of chronic kidney disease [13]. Therefore, proteinuria and the SCr level at renal biopsy may reflect chronic but not active lesions, which may respond to immunosuppressive treatment, and may serve as prognostic predictors for LN.

The main objective of this study was to explore the histology of active and chronic lesions, as well as their association with proteinuria and SCr level, in patients with LN.

## Methods

### Study design, setting, and population

We retrospectively reviewed patients with LN at Okayama University Hospital. Data from 1990 to 2006 and 2007–2015 were collected from paper- and electronic-based records, respectively. Data collection was completed in 2016–2017. The enrolled patients fulfilled the 1997 American College of Rheumatology revised criteria for the classification of systemic lupus erythematosus (SLE) [14]. Patients were eligible for participation in this study if they had a histologically confirmed diagnosis of LN (class III, IV, or V) according to the International Society of Nephrology/Renal Pathology Society (ISN/RPS) classification [15]. Eligible patients were followed up from their first renal biopsy for 10 years, until December 2015.

### Clinical parameters

The following information was collected at the time of the renal biopsy, prior to treatment: age, sex, SLE disease activity index 2000, daily maximum dose of prednisolone, use of immunosuppressants, SCr and eGFR levels, urinary protein levels (g/gCr), hematuria (dipstick test > 2+ and > 5 erythrocytes per high power field), and active urine sediments. The eGFR was evaluated by the equation developed by the Japanese Society of Nephrology [16].

### Histological parameters

For all participants, the histology of the first renal biopsy sample was classified according to the ISN/RPS classification by experienced nephrologists and/or pathologists. Active glomerular lesions were defined by the presence of endocapillary hypercellularity, leukocyte infiltration, subendothelial hyaline deposits, interstitial inflammation, karyorrhexis, fibrinoid necrosis, and cellular crescent formation. Active interstitial lesions were defined by monocellular infiltration. Chronic glomerular lesions were defined by glomerular sclerosis, fibrosis adhesion, and fibrous crescent formation, whereas chronic interstitial lesions were defined by interstitial fibrosis. Arteriosclerosis was also defined by chronic lesions. Each renal biopsy sample was processed using light and immunofluorescence microscopy with standard methods of fixation and staining. For semi-quantitative analysis, the histological score was calculated as described in our previous study [17], where histological score = (0.5 × number of glomeruli with segmental lesions + 1 × number of glomeruli with global lesions)/total number of glomeruli. Interstitial lesions such as interstitial fibrosis, arteriosclerosis, and monocellular infiltration to interstitial, tubular, and vascular lesions were semi-quantitatively graded on a scale of 0, 1, 2, and 3 (absent, mild, moderate, and severe, respectively). Interstitial lesions were categorized as high- or low-grade according to a cutoff score of 2.

### Outcomes

The primary outcome measures were the urinary protein and SCr levels. The secondary outcome measure was the cumulative 10-year renal survival rates from the date of the renal biopsy. The renal endpoint was defined as > 40% decline in the eGFR.

### Statistical analysis

Statistical analyses were performed using JMP® 14 software (SAS Institute Inc., Cary, NC, USA) and STATA v15 (StataCorp, College Station, TX, USA). All statistical tests were 2-sided. *p* < 0 .05 was considered to indicate a statistically significant difference. Complete case analyses were performed, excluding patients with missing clinical data at the time of the first biopsy. The descriptive statistics are expressed as the mean and standard deviation (SD) for continuous variables, and as n (%) for categorical variables. The cumulative renal survival rates were calculated using Kaplan-Meier analysis. We censored patients that did not reach the renal endpoint when they completed the 10-year follow-up or at the date of the last recorded visit until December 31, 2015. We calculated the number of patients at risk for reaching the endpoint from the date of the renal biopsy. Patients were grouped according to urinary protein levels and the eGFR, and survival rates were assessed using log-rank tests.

Subsequently, multiple linear regression (ordinary least squares regression) analysis was performed to explore whether the histological findings contributed to urinary protein and SCr levels. The primary dependent variables were urinary protein and SCr levels at the time of renal biopsy, which were recorded as continuous variables, and the candidate variable was the renal histological score. Urinary protein levels were log-transformed to fulfill the assumption of a normal distribution of the residuals. To address multicollinearity, which was assessed using the variance inflation factor (VIF) [18, 19], we analyzed our data as two separate models excluding highly correlated covariates. We performed multiple linear regression analysis including age and sex for sensitivity analysis.

## Results

### Patient characteristics at renal biopsy

From a total of 158 patients with LN, 119 patients with ISN/RPS class III, IV, and/or V were enrolled in the present study after eliminating 11 patients with class I, II, or VI; 20 patients with a lack of clinical data; and eight patients who underwent a re-biopsy. Patient characteristics are shown in Table 1. The mean age of the patients was 45 years, and 79% were female. The mean SCr level and eGFR were 0.87 mg/dl and 77.3 ml/min/m^2^, respectively, at the time of the first renal biopsy. The mean urinary protein level was 3.00 g/gCr. Forty-six (39%) patients were treated with prednisolone alone and the others were treated with concomitant immunosuppressants for remission induction. Renal histology revealed that class IV (71%) was the most common type, followed by class III (17%) and class V (13%). The mean (SD) scores of each histological lesion were as follows: endocapillary proliferation, 0.26 (0.30); karyorrhexis, 0.06 (0.12); fibrinoid necrosis, 0.08 (0.14); rupture of the glomerular basement membranes, 0.01 (0.03); extracapillary proliferation, 0.05 (0.10); wire loop lesions, 0.14 (0.27); hyaline deposits, 0.02 (0.06); membranous lesions, 0.11 (0.28); glomerular sclerosis, 0.11 (0.16); fibrous adhesions, 0.04 (0.07); and fibrous crescents, 0.01 (0.04). The proportions of monocellular infiltration, interstitial fibrosis, and arteriosclerosis with histological grade ≥ 2 were 43 (36%), 41 (34%), and 23 (19%), respectively. The Pearson’s correlation coefficient for each histological lesion is presented in Supplementary Table 1.

**Table 1:** Patient characteristics at renal biopsy

Characteristic	Total		Missing
	*n* = 119		(%)
Age, years	45	(±16)	–
Sex, female, n (%)	94	(79)	–
Observation period, days	2958	(±2584)	–
SLEDAI-2 K score	16	(±6)	53.8
ISN/RPS classification			
Class III, n (%)	20	(17)	–
Class III + V, n (%)	2	(10)	–
Class IV, n (%)	84	(71)	–
Class IV + V, n (%)	7	(8)	0.8
Class V, n (%)	15	(13)	–
Max dose of PSL, mg/day	37	(±15)	5.0
Immunosuppressive therapy for remission induction			4.2
PSL alone, n (%)	46	(39)	
Cyclophosphamide, n (%)	31	(26)	–
Tacrolimus or cyclosporine, n (%)	22	(19)	–
Mycophenolate mofetil, n (%)	13	(11)	–
Others, n (%)	2	(2)	–
Serum creatinine, mg/dl	0.87	(±0.51)	–
eGFR, ml/min/m^2^	77.3	(±31.0)	–
Urinary protein, g/gCr	3.00	(±2.78)	–
Hematuria (scale > 2+), n (%)	52	(44)	2.5
Active urinary sediment, n (%)	77	(65)	4.2

Data are presented as the mean and standard deviation in brackets. *SLEDAI-2 K* Systemic Lupus Erythematosus Disease Activity Index 2000; *ISN/RPS* International Society of Nephrology/Renal Pathology Society; *PSL* Prednisolone; *eGFR* Estimated glomerular filtration rate

**Fig. 1:**
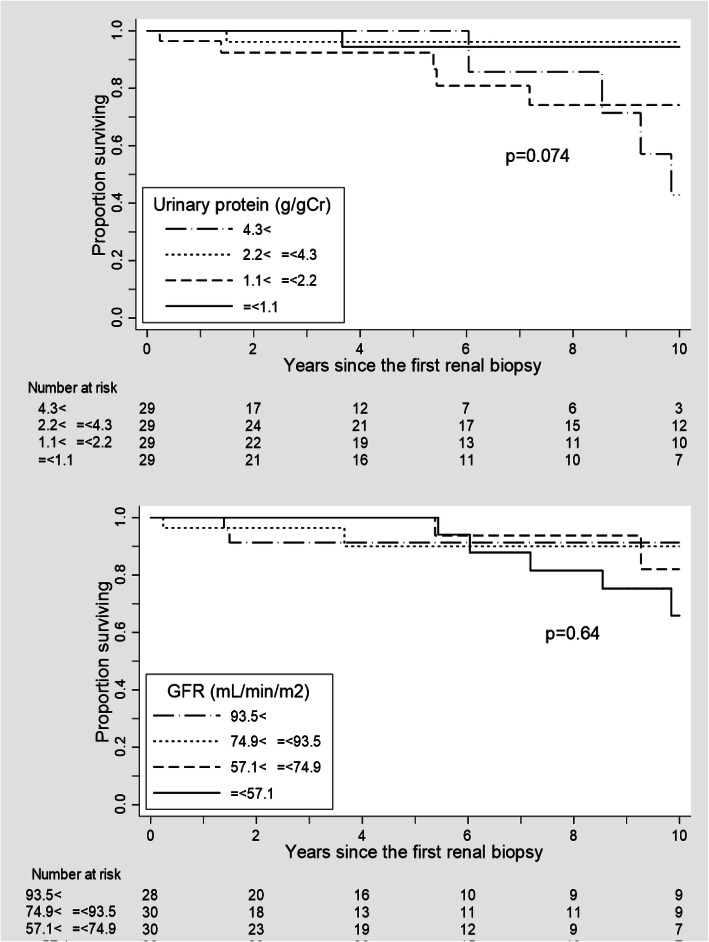
Cumulative renal survival rate of the enrolled patients, stratified according to (**a**) urinary protein levels and (**b**) estimated glomerular filtration rate (eGFR) at renal biopsy

During the mean observation period of 1931 days, 11 (9.2%) patients experienced a > 40% decrease in eGFR. There was no significant difference in the renal 10-year survival rate among the patients divided into four categories of urinary protein levels (Fig. 1 (a): log-rank test, *p* = 0.37). Similarly, the renal survival rate did not differ among patients stratified by SCr levels (Fig. 1 (b): log-rank test, *p* = 0.88).

### Explanatory histological variables for urinary protein levels (log-transformed)

Multiple regression analysis was used to explore the explanatory variables related to urinary protein levels (Table 2, Model 1, mean VIF = 3.08). Wire loop lesions emerged as an independent explanatory variable (β-coefficient [β]: 1.08 and 95% confidence interval [CI]: 0.33 to 1.82). Multicollinearity was confirmed between monocellular infiltration (VIF = 10.22) and interstitial fibrosis (VIF = 10.29), and between karyorrhexis (VIF = 4.14) and fibrinoid necrosis (VIF = 4.29). Therefore, fibrinoid necrosis and monocellular infiltration were excluded from subsequent multiple regression analysis (Table 2, Model 2, mean VIF = 1.40). In Model 2, wire loop lesions (β: 1.09 and 95% CI: 0.35 to 1.83) were also detected as an independent explanatory variable for proteinuria. When fibrinoid necrosis and interstitial fibrosis were excluded from the analysis in Model 3, wire loop lesions (β: 1.00 and 95% CI: 0.28 to 1.71) were still detected as an independent explanatory variable (Table 2, Model 3, mean VIF = 1.40). Sensitivity analysis including age and sex showed that only wire loop lesions were significantly related to proteinuria (β: 1.10 and 95% CI: 0.35 to 1.85 in Model 2; β: 1.01 and 95% CI: 0.29 to 1.73 in Model 3) (Supplementary Table 2).

**Table 2:** Multiple regression analysis of log-transformed urinary protein levels at renal biopsy and histological variables

Variable	Model 1	Model 2	Model 3
	Coeff [95% CI]	Coeff [95% CI]	Coeff [95% CI]
Active lesions			
Endocapillary proliferation	0.66 [− 0.12 to 1.44]	0.64 [− 0.13 to 1.41]	0.67 [− 0.10 to 1.43]
Karyorrhexis	0.93 [−2.11 to 3.97]	0.87 [− 1.14 to 2.87]	0.84 [− 1.16 to 2.83]
Fibrinoid necrosis	−0.12 [− 2.82 to 2.58]		
Rupture of glomerular basement membranes	2.34 [−5.52 to 10.21]	2.26 [−4.48 to 9.00]	2.14 [−4.60 to 8.87]
Extracapillary proliferation	1.79 [−0.63 to 4.22]	1.77 [−0.62 to 4.16]	1.84 [− 0.54 to 4.22]
Wire loop lesions	1.08 [0.33 to 1.82]	1.09 [0.35 to 1.83]	1.00 [0.28 to 1.71]
Hyaline deposits	1.18 [−2.65 to 5.00]	1.12 [−2.65 to 4.89]	1.39 [− 2.34 to 5.12]
Membranous lesions	0.64 [−0.07 to 1.35]	0.67 [− 0.02 to 1.35]	0.67 [− 0.01 to 1.36]
Monocellular infiltration (category)	− 0.25 [− 1.48 to 0.98]		− 0.07 [− 0.53 to 0.39]
Chronic lesions			
Glomerular sclerosis	1.13 [− 0.28 to 2.54]	1.15 [− 0.24 to 2.54]	1.13 [− 0.24 to 2.49]
Fibrous adhesion	− 0.40 [− 3.41 to 2.62]	−0.35 [− 3.33 to 2.63]	−0.25 [− 3.21 to 2.71]
Fibrous crescents	1.63 [− 3.93 to 7.19]	1.60 [− 3.76 to 6.95]	1.80 [− 3.56 to 7.15]
Interstitial fibrosis (category)	0.23 [− 1.01 to 1.47]	0.00 [− 0.47 to 0.47]	
Arteriosclerosis (category)	0.15 [− 0.38 to 0.68]	0.13 [− 0.39 to 0.64]	0.17 [− 0.35 to 0.68]

Proteinuria was log-transformed to obtain a closer approximation to normal distribution. Covariates; Model 1: all independent explanatory variables; Model 2: explanatory variables excluding fibrinoid necrosis and monocellular infiltration; Model 3: explanatory variables excluding fibrinoid necrosis and interstitial fibrosis. *Coeff* β-Coefficient; *CI* Confidential interval

### Explanatory histological variables for serum creatinine levels

Similarly to the analysis for urinary protein level, multiple regression analysis using all the histological variables was performed to explore the explanatory variables related to the SCr level (Table 3, Model 1, Mean VIF = 3.08). Hyaline deposits (β: − 1.84 and 95% CI: − 3.64 to − 0.04) and glomerular sclerosis (β: 1.10 and 95% CI: 0.43 to 1.76) emerged as independent explanatory variables in Model 1. After excluding fibrinoid necrosis and monocellular infiltration in Model 2, and fibrinoid necrosis and interstitial fibrosis in Model 3, glomerular sclerosis was still an independent explanatory variable (Table 3, mean VIF = 1.40 in Model 2 and mean VIF = 1.40 in Model 3). Sensitivity analysis including age and sex showed that glomerular sclerosis was a constant independent explanatory variable for SCr (β: 1.01 and 95% CI: 0.37 to 1.66 in Model 2; β: 1.01 and 95% CI: 0.38 to 1.64 in Model 3) (Supplementary Table 3).

**Table 3:** Multiple regression analysis of serum creatinine levels at renal biopsy and histological variables

Variable	Model 1	Model 2	Model 3
	Coeff [95% CI]	Coeff [95% CI]	Coeff [95% CI]
Active lesions			
Endocapillary proliferation	0.15 [− 0.22 to 0.51]	0.16 [− 0.20 to 0.52]	0.16 [− 0.2 to 0.52]
Karyorrhexis	0.08 [− 1.34 to 1.51]	0.20 [− 0.74 to 1.15]	0.23 [− 0.71 to 1.17]
Fibrinoid necrosis	0.18 [− 1.08 to 1.45]		
Rupture of glomerular basement membranes	0.16 [− 3.54 to 3.85]	0.34 [− 2.83 to 3.52]	0.39 [− 2.79 to 3.56]
Extracapillary proliferation	0.37 [− 0.77 to 1.51]	0.40 [− 0.73 to 1.52]	0.42 [− 0.7 to 1.54]
Wire loop lesions	0.13 [− 0.22 to 0.48]	0.12 [− 0.22 to 0.47]	0.08 [− 0.26 to 0.42]
Hyaline deposits	−1.84 [− 3.64 to − 0.04]	− 1.78 [− 3.56 to − 0.00]	− 1.67 [− 3.43 to 0.09]
Membranous lesions	−0.17 [− 0.50 to 0.17]	−0.19 [− 0.51 to 0.13]	−0.15 [− 0.48 to 0.17]
Monocellular infiltration (category)	0.22 [− 0.36 to 0.80]		0.26 [0.04 to 0.47]
Chronic lesions			
Glomerular sclerosis	1.10 [0.43 to 1.76]	1.08 [0.43 to 1.74]	1.07 [0.43 to 1.72]
Fibrous adhesion	0.73 [−0.69 to 2.14]	0.68 [−0.72 to 2.08]	0.79 [− 0.61 to 2.19]
Fibrous crescents	−1.30 [− 3.91 to 1.31]	−1.31 [− 3.84 to 1.21]	− 1.32 [− 3.84 to 1.21]
Interstitial fibrosis (category)	0.06 [− 0.52 to 0.65]	0.27 [0.05 to 0.49]	
Arteriosclerosis (category)	− 0.04 [− 0.29 to 0.21]	−0.02 [− 0.26 to 0.23]	− 0.02 [− 0.26 to 0.23]

Covariates; Model 1: all independent explanatory variables; Model 2: explanatory variables excluding fibrinoid necrosis and monocellular infiltration; Model 3: explanatory variables excluding fibrinoid necrosis and interstitial fibrosis. *Coeff* β-Coefficient; *CI* Confidential interval

## Discussion

In this study, we explored the associations between histological findings and urinary protein and SCr levels at renal biopsy. The urinary protein level was reflected only in wire loop lesions, whereas the SCr level was only correlated with glomerular sclerosis.

We could not confirm urinary protein and SCr levels as predictive factors for the renal outcome of patients with LN. As several previous reports showed that urinary protein and SCr levels are the main predictors for renal outcome [5–8], there is no doubt that they may serve as predictors of renal prognosis. As the patients in the present study had less severe disease and a shorter observation period than previously reported, urinary protein and SCr levels were unable to predict renal prognosis in the present study.

Urinary protein level mainly reflected wire loop, but not endocapillary or extracapillary, proliferative lesions in our study. Our previous report showed that only extracapillary proliferation was associated with poor renal predictors in active lesion [17]. To the best of our knowledge, the association between histological findings and proteinuria has not been fully established in patients with LN. Previous reports showed that the urinary protein level was related to the activity index [20, 21], while another report showed that proteinuria was not correlated with activity and chronicity indices [22]. A previous study revealed that proteinuria was correlated with hyaline deposition [12]. Considering these results, the application of urinary protein levels as a biomarker of treatment response for active LN may be challenging.

The SCr level was the only factor indicative of glomerular sclerosis in our study. This finding is supported by previous studies showing that the SCr level mainly reflects chronicity in LN [12, 17, 22], and is correlated with renal interstitial lesions, sclerotic glomeruli, and tubular atrophy [23, 24]. Although the SCr level is a promising biomarker for renal prognosis in patients with LN, it was only associated with chronic lesions. Therefore, the SCr level, similar to the urinary protein level, may not be applicable as a biomarker of treatment response in patients with LN with active lesions.

The present study had certain limitations. Firstly, this is a cross-sectional study performed at renal biopsy; therefore, we were unable to evaluate whether urinary protein and SCr levels directly reflect the treatment response of active lesions. However, we concluded that SCr was unlikely to recover to a normal range if glomerular sclerosis was present. Secondly, we did not evaluate treatment effects related to outcomes. Treatment may be adjusted according to not only histological findings, but also urinary protein and renal function. As our patients exhibited a better renal outcome than previous reports [6], we may have underestimated the predictive power of proteinuria and renal function for renal outcome.

## Conclusions

The present study revealed that urinary protein and SCr levels did not quantitatively reflect active lesions in LN. Therefore, they may not be adequate biomarkers for measuring the response to remission induction therapy in patients with LN. Comparing the changes in candidate biomarkers and histological findings before and after treatment may aid the identification of potential biomarkers for monitoring the treatment response.

## Supplementary information

**Additional file 1 Supplementary Table 1.** Correlation matrix of the histological findings. * *p* < 0.05. **Supplementary Table 2.** Multiple regression analysis of log-transformed urinary protein levels at renal biopsy and histological variables. Proteinuria was log-transformed to obtain a closer approximation to normal distribution. Covariates; Model 1: All independent explanatory variables; Model 2: Independent explanatory variables excluding fibrinoid necrosis and monocellular infiltration; Model 3: Independent explanatory variables excluding fibrinoid necrosis and interstitial fibrosis. 95% confidence intervals in brackets. * *p* < 0.05, ** *p* < 0.01, *** *p* < 0.001. **Supplementary Table 3.** Multiple regression analysis of serum creatinine levels at renal biopsy and histological scores**.** Covariates; Model 1: All independent explanatory variables; Model 2: Independent explanatory variables excluding fibrinoid necrosis and monocellular infiltration; Model 3: Independent explanatory variables excluding fibrinoid necrosis and interstitial fibrosis. 95% confidence intervals in brackets. * *p* < 0.05, ** *p* < 0.01, *** *p* < 0.001.

## Data Availability

The datasets used and/or analyzed during the current study are available from the corresponding author on reasonable request.

## Abbreviations

LN: Lupus nephritis
SCr: Serum creatinine
eGFR: Estimated glomerular filtration rate
ISN/RPS: International society of nephrology/renal pathology society
SD: Standard deviation
VIF: Variance inflation factor
β: β-Coefficient
CI: Confidence interval
