# Supplementary material for: Association of explanatory histological findings and urinary protein and serum creatinine levels at renal biopsy in lupus nephritis: a cross-sectional study
Source: BMC Nephrol. 2020 Jun 1;21:208. doi: 10.1186/s12882-020-01868-9 (PMC7268330; doi:10.1186/s12882-020-01868-9)
Supplement: Supplementary file 1 — Additional file 1 Supplementary Table 1. Correlation matrix of the histological findings. * p < 0.05. Supplementary Table 2. Multiple regression analysis of log-transformed urinary protein levels at renal biopsy and histological variables. Proteinuria was log-transformed to obtain a closer approximation to normal distribution. Covariates; Model 1: All independent explanatory variables; Model 2: Independent explanatory variables excluding fibrinoid necrosis and monocellular infiltration; Model 3: Independent explanatory variables excluding fibrinoid necrosis and interstitial fibrosis. 95% confidence intervals in brackets. * p < 0.05, ** p < 0.01, *** p < 0.001. Supplementary Table 3. Multiple regression analysis of serum creatinine levels at renal biopsy and histological scores. Covariates; Model 1: All independent explanatory variables; Model 2: Independent explanatory variables excluding fibrinoid necrosis and monocellular infiltration; Model 3: Independent explanatory variables excluding fibrinoid necrosis and interstitial fibrosis. 95% confidence intervals in brackets. * p < 0.05, ** p < 0.01, *** p < 0.001. [file 12882_2020_1868_MOESM1_ESM.rtf]

Active lesions	Endocapillary proliferation	Karyorrhexis	Fibrinoid necrosis	Rupture of glomerular
basement membranes	Extracapillary proliferation	Wire loop lesion	Hyaline deposits	Membranous	Monocellular infiltration
(category)	
Endocapillary proliferation	1.000	
Karyorrhexis	0.569*	1.000	
Fibrinoid necrosis	0.431*	0.801*	1.000	
Rupture of glomerular basement membranes	0.031	0.189*	0.464*	1.000	
Extracapillary proliferation	0.224*	0.456*	0.497*	0.377*	1.000	
Wire loop lesion	-0.087	-0.123	-0.136	-0.094	-0.089	1.000	
Hyaline deposits	0.036	0.154	0.163	0.113	0.117	0.240*	1.000	
Membranous	-0.212*	-0.144	-0.138	-0.141	-0.180	-0.060	-0.079	1.000	
Monocellular infiltration
(category)	0.022	0.096	0.080	0.051	0.180	-0.069	0.184*	-0.142	1.000	
Supplementary table1. Matrix of Correlations matrix of the histological findings.
Chronic lesions	Glomerular sclerosis	Fibrous 
adhesion	Fibrous 
crescents	Interstitial fibrosis
(category)	Arterio-
sclerosis
(category)	
Glomerular sclerosis	1.000	
Fibrous adhesion	0.146	1.000	
Fibrous crescents	0.122	0.394*	1.000	
Interstitial fibrosis
(category)	0.472*	0.212*	0.257*	1.000	
Arteriosclerosis
(category)	0.286*	0.036	-0.063	0.313*	1.000	
	
* p < 0.05.	

Supplementary table 2. Multiple regression analysis of log-transformed urinary protein levels at renal biopsy and histological variables.
	Model1		Model2		Model3	
Variables	â coefficient [95�“CI]		â coefficient [95�“CI]		â coefficient [95�“CI]	
Age	0.00	[-0.01 to 0.02]		0.00	[-0.01 to 0.02]		0.00	[-0.01 to 0.02]	
Sex	-0.08	[-0.60 to 0.43]		-0.09	[-0.60 to 0.42]		-0.03	[-0.52 to 0.46]	
Active lesions						
	Endocapillary proliferation	0.69	[-0.11 to 1.48]		0.67	[-0.12 to 1.45]		0.70	[-0.08 to 1.48]	
	Karyorrhexis	0.81	[-2.30 to 3.92]		0.78	[-1.28 to 2.83]		0.76	[-1.29 to 2.81]	
	Fibrinoid necrosis	-0.09	[-2.81 to 2.64]							
	Rupture of glomerular
basement membranes	2.54	[-5.52 to 10.59]		2.52	[-4.42 to 9.46]		2.26	[-4.66 to 9.18]	
	Extracapillary proliferation	1.72	[-0.75 to 4.20]		1.70	[-0.73 to 4.14]		1.80	[-0.62 to 4.23]	
	Wire loop lesion	1.09**	[0.33 to 1.85]		1.10**	[0.35 to 1.85]		1.01**	[0.29 to 1.73]	
	Hyaline deposits	1.17	[-2.70 to 5.03]		1.11	[-2.70 to 4.92]		1.37	[-2.40 to 5.13]	
	Membranous	0.65	[-0.07 to 1.37]		0.68	[-0.01 to 1.38]		0.68	[-0.02 to 1.38]	
	Monocellular infiltration
(category)	-0.25	[-1.50 to 0.99]					-0.08	[-0.55 to 0.38]	
Chronic lesions						
	Glomerular sclerosis	1.13	[-0.30 to 2.56]		1.15	[-0.27 to 2.56]		1.10	[-0.27 to 2.48]	
	Fibrous adhesion	-0.44	[-3.50 to 2.63]		-0.39	[-3.42 to 2.63]		-0.26	[-3.26 to 2.75]	
	Fibrous crescents	1.56	[-4.05 to 7.18]		1.52	[-3.90 to 6.93]		1.72	[-3.69 to 7.14]	
	Interstitial fibrosis
(category)	0.23	[-1.03 to 1.48]		-0.01	[-0.49 to 0.47]				
	Arteriosclerosis
(category)	0.11	[-0.45 to 0.68]		0.09	[-0.46 to 0.64]		0.12	[-0.43 to 0.67]	
Proteinuria was log-transformed to obtain a closer approximation to normal distribution. Covariates; Model 1: All independent explanatory variables; Model 2: Independent explanatory variables excluding fibrinoid necrosis and monocellular infiltration; Model 3: Independent explanatory variables excluding fibrinoid necrosis and interstitial fibrosis. 95% confidence intervals in brackets. * p < 0.05, ** p < 0.01, *** p < 0.001.
Supplementary table 3. Multiple regression analysis of serum creatinine levels at renal biopsy and histological scores.
	Model1		Model2		Model3	
Variables	â coefficient [95�“CI]		â coefficient [95�“CI]		â coefficient [95�“CI]	
Age	0.01*	[0.00 to 0.02]		0.01*	[0.00 to 0.02]		0.01**	[0.00 to 0.02]	
Sex	-0.01	[-0.24 to 0.23]		-0.00	[-0.23 to 0.23]		0.02	[-0.21 to 0.24]	
Active lesions						
	Endocapillary proliferation	0.21	[-0.15 to 0.57]		0.23	[-0.13 to 0.58]		0.22	[-0.14 to 0.57]	
	Karyorrhexis	-0.14	[-1.55 to 1.28]		0.07	[-0.87 to 1.01]		0.10	[-0.83 to 1.04]	
	Fibrinoid necrosis	0.28	[-0.96 to 1.52]							
	Rupture of glomerular
basement membranes	0.10	[-3.57 to 3.77]		0.42	[-2.74 to 3.59]		0.43	[-2.72 to 3.58]	
	Extracapillary proliferation	0.33	[-0.80 to 1.45]		0.36	[-0.75 to 1.48]		0.39	[-0.72 to 1.49]	
	Wire loop lesion	0.13	[-0.21 to 0.48]		0.13	[-0.22 to 0.47]		0.10	[-0.23 to 0.43]	
	Hyaline deposits	-1.86*	[-3.61 to -0.10]		-1.79*	[-3.53 to -0.05]		-1.73*	[-3.45 to -0.02]	
	Membranous	-0.16	[-0.48 to 0.17]		-0.18	[-0.49 to 0.14]		-0.15	[-0.46 to 0.17]	
	Monocellular infiltration
(category)	0.18	[-0.38 to 0.75]					0.23*	[0.02 to 0.44]	
Chronic lesions						
	Glomerular sclerosis	1.02**	[0.37 to 1.67]		1.01**	[0.37 to 1.66]		1.01**	[0.38 to 1.64]	
	Fibrous adhesion	0.78	[-0.61 to 2.18]		0.74	[-0.64 to 2.12]		0.83	[-0.53 to 2.20]	
	Fibrous crescents	-1.38	[-3.94 to 1.18]		-1.44	[-3.91 to 1.03]		-1.45	[-3.92 to 1.01]	
	Interstitial fibrosis
(category)	0.07	[-0.50 to 0.64]		0.24*	[0.02 to 0.46]				
	Arteriosclerosis
(category)	-0.14	[-0.40 to 0.12]		-0.12	[-0.37 to 0.13]		-0.13	[-0.38 to 0.12]	
Covariates; Model 1: All independent explanatory variables; Model 2: Independent explanatory variables excluding fibrinoid necrosis and monocellular infiltration; Model 3: Independent explanatory variables excluding fibrinoid necrosis and interstitial fibrosis. 95% confidence intervals in brackets. * p < 0.05, ** p < 0.01, *** p < 0.001.
